# Trends in the prevalence, prenatal diagnosis, and outcomes of births with chromosomal abnormalities: a hospital-based study in Zhejiang Province, China during 2014–2020

**DOI:** 10.1186/s13023-022-02594-1

**Published:** 2022-12-22

**Authors:** Xinning Chen, Dan Lin, Yinghui Ye, Xiaohui Zhang, Danqing Chen

**Affiliations:** 1 Department of Obstetric, Women’s Hospital, Zhejiang University School of Medicine, Hangzhou, Zhejiang Province China; 2 Department of Women’s Health, Women’s Hospital, Zhejiang University School of Medicine, Hangzhou, Zhejiang Province China; 3 Key Laboratory of Reproductive Genetics, School of Medicine, Women’s Hospital, Zhejiang University, Hangzhou, Zhejiang China

## Abstract

**Background:**

To investigate the prevalence and prenatal diagnosis rate of chromosomal abnormalities (CA) in Zhejiang Province, China.

**Methods:**

We estimated the annual changes in the detected prevalence of CA and prenatal diagnosis rate among 681,590 births in Zhejiang Province, China, between 2014 and 2020. Data were derived from the provincial birth defects surveillance system, which represents 30% of annual births in Zhejiang Province. The effect of maternal age was also evaluated.

**Results:**

The detected prevalence of sex chromosomal abnormalities (1.70–7.30 per 10,000 births, *P*_trend_ < 0.001) and microdeletion and microduplication (0.30–6.81 per 10,000 births, *P*_trend_ < 0.001) gradually increased, contributing to an upward trend in overall CA (12.09–39.22 per 10,000 births). The diagnosis rate before 22 gestational weeks constantly increased from 20.8 to 70.1% for trisomy 21 (*P*_trend_ = 0.003). The prevalence rate ratio for maternal age of ≥ 35 years was higher than that for maternal age of 25–29 years for trisomy 21 (5.40, 95% confidence interval [CI] 4.59–6.35) and sex chromosomal abnormalities (3.28, 95% CI 2.48–4.33).

**Conclusions:**

The rising prevalence of CA in China may be attributable to the elevated maternal age and the innovation of prenatal diagnosis tools, Thus, studies should pay attention to the rare CA that were previously ignored, and select rational screening tools.

**Supplementary Information:**

The online version contains supplementary material available at 10.1186/s13023-022-02594-1.

## Background

Chromosomal abnormalities (CA) are common congenital abnormalities in human embryos and newborns, indicating numerical and structural chromosomal aberrations. The total prevalence of CA ranges from 48 to 90 per 10,000 births, depending on several factors such as race, observational time, and regional differences [1, 2]. CA is a major cause of early miscarriages and stillbirths, and is strongly associated with multiple congenital anomalies, growth failure, and neurodevelopmental disorders in live births [3, 4].

Research revealed an increasing trend of CA in most parts of the world, especially for numerical CA [5–8]. This is mainly attributed to the rapid development of prenatal diagnosis technology and efficient screening programs, as well as elevated childbearing age [9, 10]. The combination of serum tests and nuchal translucency (NT), use of non-invasive prenatal testing (NIPT), and improved cytogenetic and molecular diagnostic techniques have paved the way for more than 90% prenatal identification of aneuploidies and a large proportion of sex chromosome anomalies (SCA) [11–13]. There is also a well-established association between risk for aneuploids and advanced maternal age [1], which partially explains the elevated prevalence of CA worldwide [2, 8]. In addition, sperm contribution and environmental factors influence the occurrence of CA [3].

China has a high burden of CA due to its large population, especially given the increasing maternal age with the adjustment of birth policies [10]. Screening for and prenatal confirmation of CA have also developed rapidly in China. Our previous study revealed an increased prevalence of trisomy 21 in Zhejiang Province, located in eastern China [10]. However, several previous studies have focused on the efficacy of screening methods and calculated the prevalence of CA in live births or were restricted to numerical CA. Zhejiang Province has a well-established hospital-based birth defect monitoring system that routinely collects CA information from pregnancy loss, stillbirths, and newborns. This study provides insights into the comprehensive epidemiology, prenatal diagnosis, and estimation of birth outcomes of CA in Zhejiang, as well as updates of our previous findings, and may provide a reference for CA prevention and intervention in other developing countries.

## Methods

### Study population and data source

Data were retrieved from the provincial birth defect surveillance system in Zhejiang Province, China. Ninety delivery hospitals across 30 regions are listed in this surveillance system, representing 30% of the annual births in the province. All births with CA diagnosed prenatally or within 7 days after birth were captured in this system. A questionnaire was administered by the medical staff in the surveillance hospitals to collect information on maternal characteristics, risk factors, and diagnosis of birth defects. The Women’s Hospital, School of Medicine, Zhejiang University is the provincial prenatal diagnosis centre, and is responsible for the quality of diagnosis data and the surveillance system registry. All cases were confirmed and referenced using the International Statistical Classification of Diseases and Related Health Problems, 10th Revision (ICD-10) codes, and were reported online. Quality control is routinely performed at the hospital, regional, and provincial levels.

We enrolled patients with CA from this system between January 2014 and December 2020. Live births, early pregnancy loss before 22 gestational weeks (GW), stillbirths at or after 22 GW, and termination of pregnancy due to foetal anomalies (TOPFA) at any gestation were included in this study. The earliest gestational ages of recorded spontaneous pregnancy loss and TOPFA were 13 and 15 GW, respectively. This study was approved by the Ethics Committee of Women’s Hospital, Zhejiang University School of Medicine (approval number: 2018KY036).

### Screening strategy and case ascertainment

Pregnant women in Zhejiang Province routinely undergo prenatal screening, comprising the first-trimester combined test (NT ultrasound and maternal serum test) or the second-trimester triple test (maternal serum test of hCG (human chorionic gonadotropin), AFP (alpha-fetoprotein) and uE3 (unconjugated estriol)). First- and second-trimester screening can be performed independently or in combination. Women identified as high-risk by first-trimester screening or abnormal ultrasonographic markers are recommended for NIPT or directly for invasive testing. If the result falls within the intermediate-risk category, second-trimester screening is recommended. If there is a low-risk for CA during the first-trimester screening, no further screening is suggested. Women who missed their first-trimester combined test were also given the option to undergo NIPT. Women with advanced age (≥ 35 years) at the time of delivery, with a history of CA or birth defects are recommended to undergo NIPT or invasive testing. The final CA diagnosis is reported by a trained medical staff in hospitals entitled to make a prenatal diagnosis, based on invasive testing from chorionic villus sampling (CVS) or amniocentesis sampling. Only a small part of CA was diagnosed after delivery, from products of conception, tissue from stillbirths, or venous blood from infants. Positive cases were transferred to diagnosis centres, wherein quality control was routinely performed.

**Fig. 1:**
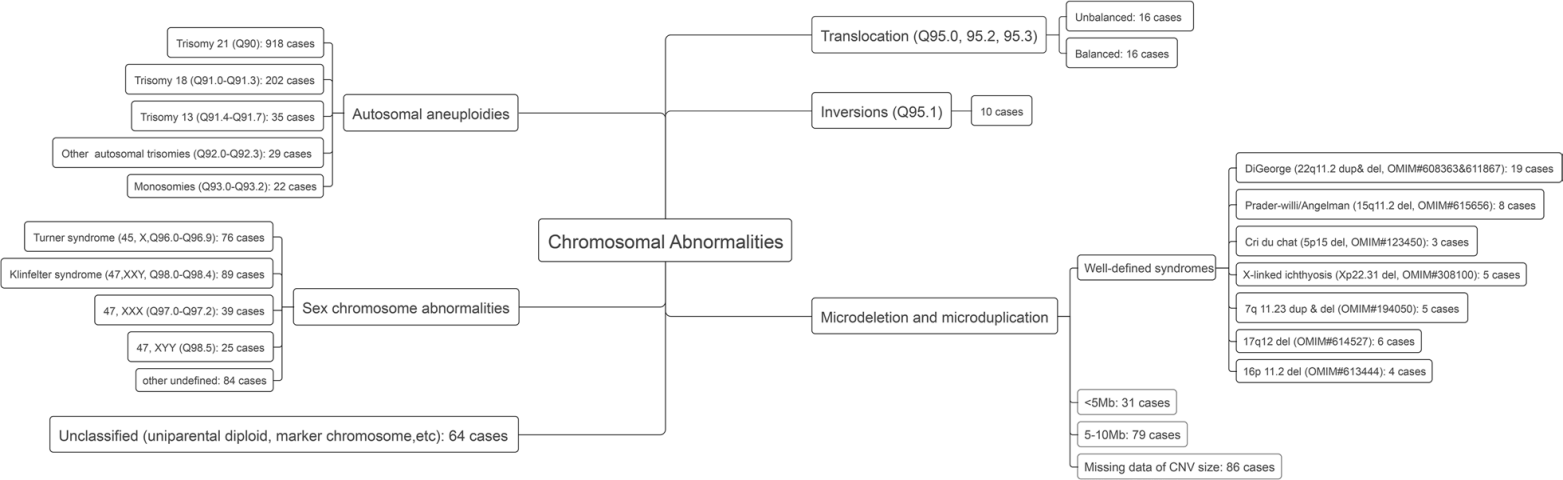
Chromosomal anomaly subtypes reported by birth defects surveillance system in Zhejiang Province, 2014–2020

High-resolution karyotypic analysis and fluorescence in situ hybridization (FISH) involves detecting numerical changes in chromosomes, especially for common aneuploidies in chromosomes 13, 18, 21, X and Y. In our study, 1206 autosomal aneuploidies, including: Down syndrome (trisomy 21, Q90), trisomy 18 (Q91.0–91.3), trisomy 13 (Q91.4–91.7), and other types of trisomy (Q92.0–92.3) and monosomy (Q93.0–93.2); 313 SCA, including Turner’s syndrome (45 X, mosaicism for X monosomy or isochromosome Xq, Q96.0–96.9), Klinefelter syndrome (47 XXY, Q98.0–98.4), 47 XXX(Q97.0–97.2), 47XYY (Q98.5), and other SCA according to ICD-10 codes (Q90–Q99) were detected (Fig. 1).

In cases with copy number variant (CNV) findings, chromosomal microarray analysis (CMA) or next-generation sequencing (NGS) provides greater information. CMA was performed using either comparative genomic hybridisation arrays or single nucleotide polymorphism using a customized high-resolution chip Affymetrix CytoScan™ HD array (Affymetrix, Santa Clara, CA, USA). The reporting threshold for the copy number was set at 500 kb with a marker count ≥ 50 for microduplications and at 200 kb with a marker count ≥ 50 for microdeletions. We reported 246 microdeletions and microduplications, 32 translocations (Q95.0, Q95.2, Q95.3) and 10 inversions (Q95.1) in normal or abnormal individuals. In addition, 64 foetuses with uniparental diploid (Q99.8, Q99.9) or marker chromosomes (Q95.4) could not be classified into any category as shown in Fig. 1. CNVs were interpreted according to the guidelines of the American College of Medical Genetics and Genomics (ACMG) [14]. Microdeletion or microduplication was defined as the submicroscopic loss or gain of a small segment of a chromosome. Well-defined microdeletion and microduplication syndromes (MMS) such as DiGeorge syndrome (OMIM# 608363 & 611867, n = 19), Prader–Willi/Angelman syndrome (PWS; OMIM# 615656, n = 8), and cri du chat syndrome (OMIM# 123450, n = 3) [15–18], as well as pathogenic, likely pathogenic, and uncertain significance variants were required to report to the surveillance system, referring to the online databases [19–21]. We included well-known MMS (n = 50), CNVs < 5 Mb (n = 31) and CNVs between 5 and 10 Mb (n = 79) for further analysis, for a wider range of anomalies.

### Statistical analyses

Maternal characteristics including education, parity, GW, and foetal prognosis (live birth, early pregnancy loss, stillbirth, TOPFA, and newborn death) were analysed as categorical variables. Maternal age was defined as the age at delivery and categorised into five groups: < 20, 20–24, 25–29, 30–34, and ≥ 35 years. The overall prevalence of CA (CA in live births, foetal death and TOPFA) and prevalence at birth (CA in live births) were calculated. The proportion of cases diagnosed of CA before 22 GW was calculated. The Cochran-Armitage test was used to analyse the prevalence and diagnosis proportions according to annual changes. We further calculated the prevalence rate ratio (PRR) and 95% confidence intervals (CI) relative to the reference maternal age group (mothers aged 25–29 years) and constructed a forest plot. Statistical analyses were performed with SPSS (version 22.0; Chicago, IL, USA) and the R project (4.0.3 for Windows). Figures were generated with GraphPad Prism 8 (SanDiego, CA, USA). Statistical significance was set at *P* < 0.05, and all *P*-values were two-tailed.

### Missing data and sensitivity analysis

In total, 86 cases with chromosomal deletions or duplications lacked information on CNV size. Therefore, we performed a sensitivity analysis by adopting different models for the missing data (Additional file 1: Fig. S1) to assess the prevalence trend of microdeletions and microduplications by year. In contrast, the missing data represented less than 1% for other variables in cases of CA, involving maternal age, education, and region, which could be ignored.

## Results

**Fig. 2:**
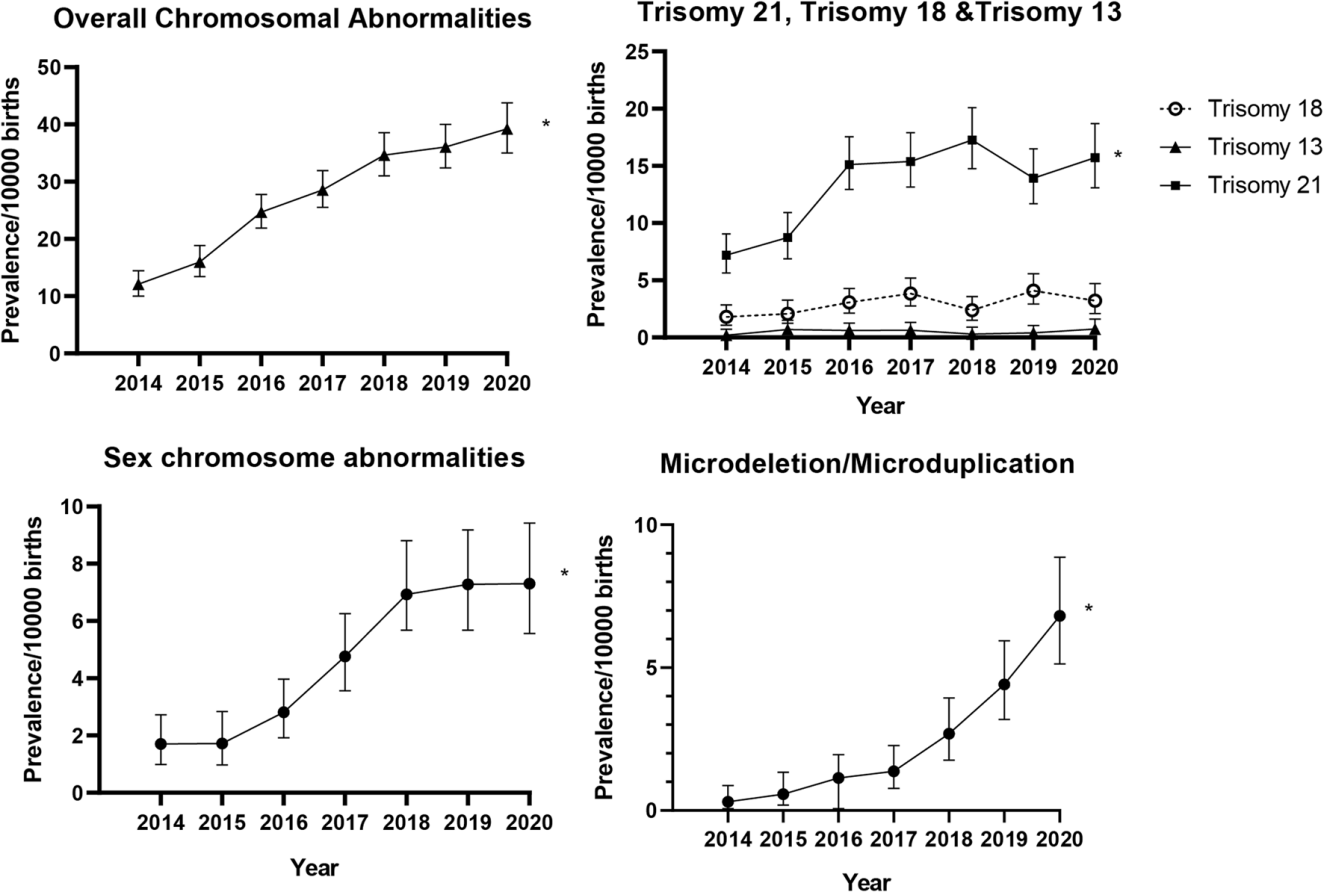
Prevalence of chromosomal abnormalities per 10,000 births in each calendar year, 2014–2020. Prevalence per 10,000 births of overall chromosomal anomalies and different subtypes of chromosomal abnormalities. *Significant differences were observed in Cochran-Armitage trend test over the years (*P*_trend_ < 0.001 for overall chromosomal anomalies, trisomy 21, sex chromosomal anomalies, microdeletion/ microduplication).

Between 2014 and 2020, there were 1857 cases of CA were diagnosed among 681,590 births. The overall prevalence of CA increased substantially from 12.09 to 39.22 per 10,000 births between 2014 and 2020 (*P*_trend_ < 0.001, Fig. 2). The prevalence of CA in live births also increased slightly, although no significant difference was observed (2.80 per 10,000 births in 2014 vs. 3.96 per 100,000 births in 2020, Additional file 2: Fig. S2). The sensitivity analysis referring to reporting centres also indicated an analogous upward trend in the prevalence in both tertiary and district hospitals, although the prevalence reported by tertiary hospitals was comparably higher (Additional file 3: Table S1).

Maternal characteristics of pregnancies with CA remained stable from 2014 to 2020 (Additional file 3: Table S2). Women who delivered foetuses with CA were more likely to be aged ≥ 30 years (n = 1165, 62.7%), primiparous (n = 1435, 77.3%), and have a higher education level (n = 1280, 68.9%). Most cases were diagnosed and reported in tertiary hospitals (n = 1661, 89.4%). The detection rate before 22 GW for overall CA was 46.9% (n = 871).

Among the 1857 cases with CA, 233 (12.5%) had one or more structural malformations detected using a sonography. The top five structural malformations associated with CA were cardiac anomalies; n = 112,), cleft lip with or without cleft palate (n = 23,), kidney malformation (n = 20, 1.2%), absence of a nasal bone (n = 18), and nuchal cystic hygroma (n = 16; Additional file 3: S3). Of these, 88.3% (n = 1640) were terminated. Among ongoing pregnancies, 11 foetal losses and 8 neonatal deaths were reported. A total of 198 (10.7%) cases were surviving neonates with CA.

### Aneuploidies

**Fig. 3:**
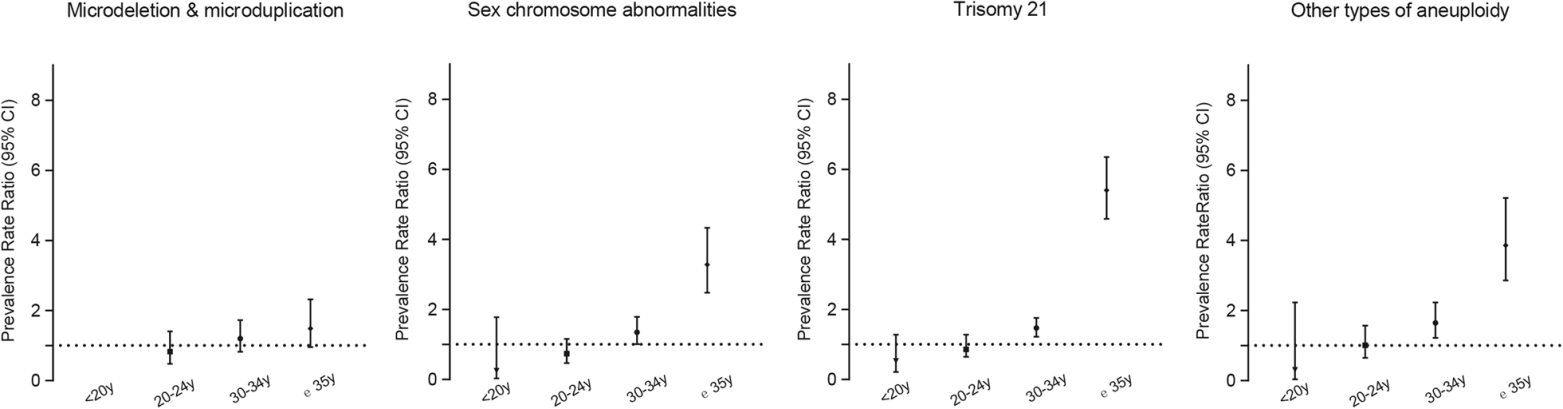
Prevalence rate ratio (95% confidence interval) of chromosomal abnormality subtypes according to maternal age compared to infants born to mothers aged 25–29. The dash line indicates significant differences

Trisomy 21 accounted for the largest proportion of cases (n = 918, 49.4%). The prevalence of trisomy 21 rose significantly in 2014–2016 but fluctuated between 2016 and 2020 (7.19 per 10,000 births in 2014 vs. 15.71 per 10,000 births in 2020), whereas trisomy 13 and trisomy 18 were maintained at low level (0.20–0.74 per 10,000 births; 1.80–3.22 per 10,000 births, respectively; Fig. 2). Trisomy 21 occurred in 42.97 of 10,000 babies born to women aged ≥ 35 years, which showed an increased risk by over five times (PRR = 5.40, 95% CI 4.59–6.35, Fig. 3), compared with the reference group (maternal age 25–29 years). The PRR also reached over three times that for other types of aneuploidies (PRR = 3.86, 95% CI 2.86–5.21, Fig. 3) compared with the reference group (maternal age 25–29 years). Typical structural anomalies associated with aneuploidies included cardiac anomalies, absence of nasal bone, atresia of digestive tract, neuroanatomic anomalies and cleft lip/cleft palate (Additional file 3: Table S3). The detection rate before 22 GW constantly increased from 20.8% in 2014 to 70.1% in 2020 for trisomy 21 (Additional file 4: Fig. S3, *P*_trend_ = 0.003). Most patients with aneuploidies (91.7% in trisomy 21; 97.9% in trisomy 18, and 94.3% in trisomy 13) were terminated.

### SCA

The prevalence of SCA (1.70–7.30 per 10,000 births, *P*_trend_ < 0.001) continually increased, especially between 2016 and 2018 (Fig. 2). Offspring born to women aged ≥ 35 years had higher risk of SCA (PRR = 3.28, 95% CI 2.48–4.33) compared with the reference group (maternal age 25–29 years) (Fig. 3). The detection rate of SCA before 22 GW increased with fluctuations (from 31.2% in 2016 to 52.5% in 2020), while the most common associated sonographic features were cardiac anomalies (n = 8) and nuchal cystic hygroma (n = 8). Of all the SCAs, 45 (14.3%) were liveborn, 4 (1.28%) resulted in a stillbirth, and 264 (84.3%) had TOPFA.

### Microdeletion/microduplication

**Fig. 4:**
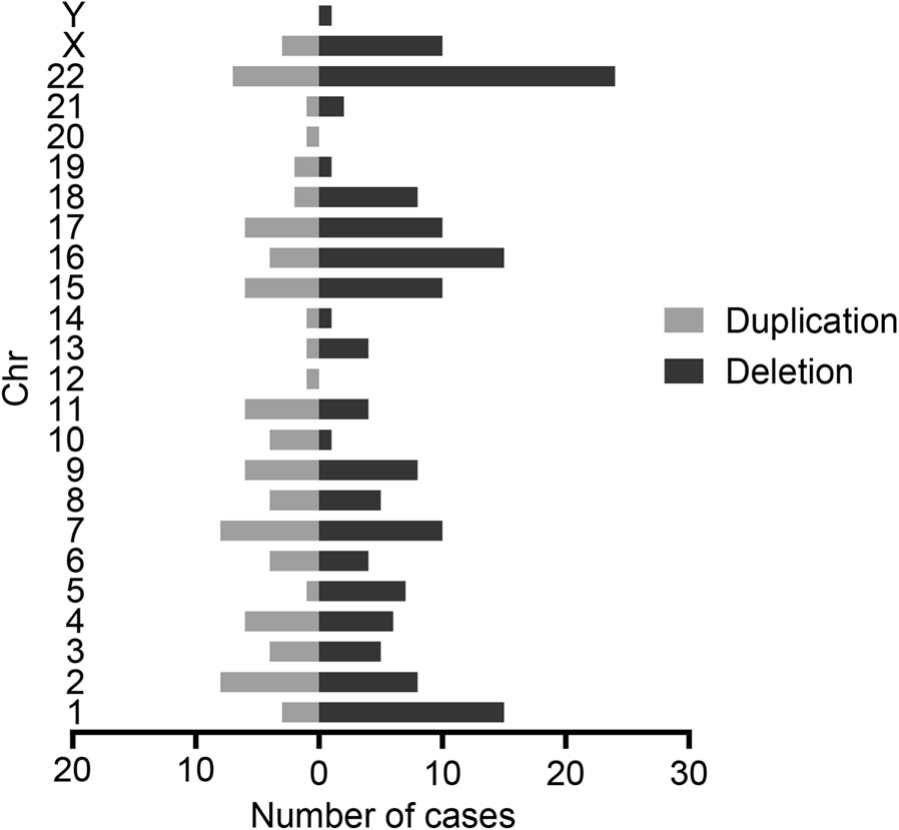
Distribution and frequency of copy number variants (CNVs) across different chromosome for microdeletion/microduplication. Black bars represent microdeletions and grey bars represent microduplications. Height represents the number of microduplication/microdeletion cases. All CNVs are summarized, including CNVs with missing size data

A total of 160 microdeletion/microduplication with CNV < 10 Mb were reported, giving a prevalence increased from to 0.30 to 6.81 per 10,000 births (*P*_trend_ < 0.001) between 2014 and 2020 (Fig. 2). The results of sensitivity analysis using different models to deal with different thresholds of CNVs confirmed a consistent upward trend in microdeletion/microduplication (Additional file 1: Fig. S1). The prevalence of microdeletion/microduplication did not change with maternal age, with a prevalence of 2.16 per 10,000 births in those aged 25–29 years, and 3.22 per 10,000 births in those aged ≥ 35 years (PRR = 1.49, 95% CI 0.96–2.32, Fig. 3). Figure 4 shows the frequency and distribution of all microdeletion/microduplication (including those missing data of CNV size) in each chromosome, with chromosome 22 having the highest frequency, followed by chromosome 16, 7 and 1. In total, 19 cases were associated with cardiac anomalies and 13 cases with kidney malformation (Additional file 3: Table S3). The detection rate of microdeletion/microduplication before 22 GW was relatively stable between 20 and 40%.

## Discussion

### Main findings

We observed an overall rising prevalence of CA, particularly in trisomy 21, SCA, and microdeletion/microduplication from 2014 to 2020 in Zhejiang Province, China. However, the prevalence of CA in live births has remained stable over time. Aneuploidies and SCA were strongly associated with maternal age, with the risk increasing by 3–5 times in women aged ≥ 35 years compared with those aged 25–29 years. Upward trends with a sharp increase in the detection proportions of CA before 22 GW, particularly at trisomy 21, were observed. Relatively, trisomy 21 and SCA had higher detection rates than microdeletion/microduplication. Overall, 88.3% of the pregnancies with CA were terminated.

### Interpretations

In our study, the upward trends in CA prevalence and changes in maternal characteristics may reflect the rapid advancement of prenatal screening and improved maternal awareness over the study period in Zhejiang Province, China, as well as changes in maternal characteristics. The first- and second-trimester screening protocols, whether integrated, sequential, or contingent, provides a high detection rate [22]. Although NIPT is regarded as a second-tier cost-efficient screening method in Zhejiang, it was reported to have ahigh sensitivity for trisomy 21 (94–100%) and trisomy 18 (over 80%) [23]. The introduction of NIPT may have contributed to the increasing prenatal screening rate, especially given its efficiency and safety. Most importantly, the beginning of the universal two-child birth policy and guidelines on NIPT were implemented by the Chinese government in 2016 [10, 24]. The comprehensive factors may partially explain the obvious increase in maternal age-related CA, as the association between aneuploidies and advanced maternal age has been well established [25–27]. Strengthening health education and genetic counseling in outpatient services also promotes the uptake of screening among women.

Comparing the overall CA prevalence worldwide is difficult due to differences in inclusion criteria. Trisomy 21, trisomy 18, and trisomy 13 comprised the largest proportion of CA cases in our study, which was consistent with the previous findings in Europe and research in other regions in China [2, 28]. EUROCAT reported an overall prevalence of 25.01 per 10,000 births for trisomy 21, 6.34/10,000 births for trisomy 18, and 2.33/10,000 births for trisomy 13 between 2013 and 2019 [2], while the United States reported a prevalence of 15 per 10,000 live births for trisomy 21 [29]. Both studies reported higher rates than the detected prevalence in our study, whereas Japan reported a similar overall prevalence (approximately 10.5/10,000 births) of trisomy 21 [30]. This disparity possibly may have resulted from the differences in maternal characteristics, surveillance systems, and screening programs. For example, in Eastern Ireland, over 30% of women studied were aged ≥ 35 years, giving a prevalence of 35.7 per 10,000 births for trisomy 21 [31], whereas in our study, women of advanced age accounted for only 13% of the participants. However, our screening program showed a satisfactory performance, with a prenatal diagnosis rate of approximately 70% for trisomy 21 before 22 GW, which is comparable with the results from Norway [32].

The prevalence of SCA is estimated to be 20–40 per 10,000 births [33], most of which are diagnosed during adolescence. According to our surveillance system, the detected prevalence of SCA was approximately 7 per 10,000 births in 2020, which was consistent with a previous study from Denmark that reported a prevalence of 172 in 275,037 pregnancies [34] based on a similar screening program. A population-based cohort study in Australia also recorded an annual prenatal prevalence of SCA of 4.4 per 10,000 births between 1986 and 2016 [35]. Combined first-trimester screening is an effective tool for detecting autosomal aneuploidies, but not for SCA. The overall positive predictive value (PPV) for SCA using NIPT was about 40–55% [36–39], whereas that for special types (e.g., 47 XXX or 47 XXY) was found to be over 80% [36], resulting in a rise in prenatal detection. Early diagnosis of SCA promotes medical-care and special education, as individuals with SCA are associated with a higher risk of comorbidity, neurocognitive deficits, and lower socioeconomic status [7].

The rising prevalence in microdeletion/microduplication has attracted increased attention, despite having an increased diagnosis rate. Currently, studies on microdeletion/microduplication remains limited, compared to those on aneuplodies or SCAs. Awareness and knowledge about microdeletion/microduplication in healthcare providers are relatively poor, especially for CNVs with uncertain significance. Previous studies about microdeletion/microduplication were mainly carried out in pregnant women with clinical indications, such as advanced maternal age, family history, and abnormal ultrasonographic findings [40], or in specific population with recurrent pregnancy loss [41]. Our study revealed a preliminary data of prevalence of microdeletion/microduplication in the general population. Data from the European congenital anomaly registry reported a prevalence of microdeletions approximately 1.27 per 10,000 between 2000 and 2006 [42], lower than that of in our study. This discrepancy may by explained by different observation periods, different detection resolutions, and reporting thresholds of CNVs. In Europe, all cases with birth defects were followed up for one year after delivery, while we only reported cases diagnosed in the first week after delivery. Furthermore, the threshold of CNVs for microdeletion/microduplication has not yet reached agreement. In Zhejiang Province, we set a backbone resolution of 200 kb and 500 kb for CMA, with increased detection sensitivity; however, the clinical validity should be considered with caution.

The rising detected prevalence of microdeletion/microduplication is mainly attributed to an increase in accumulated knowledge, and universal acceptance of invasive diagnosis after counselling. The procedure-related miscarriage rate following amniocentesis and CVS decreased [43], which is consistent with the risk in women without invasive procedures [44]. CMA technology greatly facilitates the accurate detection of microdeletion/microduplication, and overcomes the disadvantages of conventional karyotypic analysis [45]. The estimated prevalence ranged from one in 1900 births to one in 50,000 births for different types of common MMS [46–48], and the prevalence varied dramatically because of different microarray coverage and depth in different laboratories and the allocation of experienced genetic counsellors. The risk for microdeletion and microduplication did not change with increasing maternal age, and some foetuses with microdeletion/microduplication had no apparent structural anomalies. This poses challenges for identification and elucidates the low diagnosis rate before 22 GW. Long-term follow-up is recommended for these infants because possible intellectual disability, autism, and multiple malformations may occur. In addition, our data demonstrated that pathogenic CNVs occurred more frequently on chromosomes 22, 17, 16 and 1, consistent with the a previous study by Chau, et al. [40]. More information about the genotype and phenotype is needed to update the database for better interpretation of variants in future studies.

The prenatal detection rate showed no significant differences between tertiary and district hospitals (Additional file 3: Table S1). Since CA diagnosis must be performed in qualified hospitals in Zhejiang, prenatal diagnosis institutions are reasonably distributed across regions. Early detection makes early termination possible and acceptable, as early abortion is associated with a lower risk of maternal complications [49]. However, the termination of CA is influenced by the legal requirements of different countries [50, 51] and categories of CA. This requires counsellors to have a clear understanding of the pros and cons of prenatal diagnosis methods, patient preferences, and ethical assessments.

### Strengths and limitations

Our large sample size and full coverage of the CA subgroups based on the provincial surveillance system indicate that our results are robust. The hospital delivery rate is 100% in Zhejiang Province, therefore, the hospital-based surveillance data are a good reflection of the population. Most previous studies have investigated the ability of current prenatal screening methods and have focused on high-risk or referral populations. Therefore, our longitudinal study provides comprehensive insights into CA and avoided possible selection bias.

This study has several limitations. First, our surveillance system only reported the detected prevalence of abnormalities diagnosed prenatally and within 7 days after delivery, whereas a large proportion of CA were not confirmed until one year after birth or in adolescence/adulthood. Few inversions and translocations were reported by this system, and the prevalence rates of SCA and microdeletion/microduplication were underestimated to some extent. Therefore, these prevalence rates should be interpreted with caution, and further birth-cohort studies with long-term follow-ups are necessary. Second, as our surveillance system was a passive reporting database, we failed to obtain detailed pathogenicity information information for microdeletion/microduplication. Therefore, we focused on the well-known MMS, CNVs < 5 Mb and CNV5-10 Mb, in order to include complete cases. Our sensitivity analysis supported the rising trend of microdeletion and microduplication after dealing with the missing data for variant size.

## Conclusions

The prevalence of CA significantly increased between 2014 and 2020 in China, which may be explained by the advanced maternal age and the innovation of prenatal diagnosis tools. The increasing numbers of cases detected with CA proved to be a major achievement for prenatal diagnosis, and highlighted the importance of pre-test and post-test counselling, along with weighing up the benefits of early diagnosis and the costs of excessive intervention.

## Supplementary Information

**Additional file 1**: **Fig. S1**. Sensitivity analysis for prevalence of microdeletion and microduplication. Sensitivity analysis was carried out for microdeletion and microduplication, since the missing data of CNV size accounted for over 20%. $ Model 1: only cases with CNV < 5 Mb were considered in the analysis. §Model 2: only cases with CNV of 5–10 Mb were in the analysis. ‖Model 3: all cases with CNV <10 Mb as well as cases missing data of CNV size were consideredin the analysis. Significant differences were observed in the Cochran-Armitage Trend Test over the years (*P*_trend_ < 0.001 for all three models of microdeletion and microduplication).

**Additional file 2**: **Fig. S2**. Livebirth prevalence of chromosomal abnormalities per 10,000 births in each calendar year, 2014–2020 Figure Legend: Live birth prevalence per 10,000 births was calculated as live births with CA/ total births. Abbreviations: CA, chromosome abnormality.

**Additional file 3**: **Table S1**. Prevalence and prenatal diagnosis proportion of chromosomal anomalies by different reporting centres according to surveillance system. **Table S2**. Baseline characteristics of births with chromosomal abnormalities in Zhejiang Province, 2014–2020. **Table S3**. Ultrasonographic malformations associated with chromosome abnormalities.

**Additional file 4**: **Fig. S3**. Prenatal diagnosis rate before 22 gestational weeks in each calendar year: 2014–2020 *Significant differences were observed by Cochran-Armitage Trend Test over years (*P*_trend_ = 0.003 for trisomy 21, *P*_trend_ = 0.09 for sex chromosome abnormalities) Abbreviations: T21, trisomy 21.

## Data Availability

The data cannot be shared publicly because we are obligated to protect the privacy of participants within the BD surveillance system of Zhejiang Province, China. Data are available from Women’s Hospital, Zhejiang University School of Medicine, for researchers who meet the criteria for access to confidential data.

## Abbreviations

CA: Chromosomal abnormalities
NT: Nuchal translucency
NIPT: Non-invasive prenatal testing
TOPFA: Termination of pregnancy due to foetal anomalies
hCG: Human chorionic gonadotropin
AFP: Alpha-fetoprotein
CVS: Chorionic villus sampling
FISH: Fluorescence in situ hybridization
CNV: Copy number variant
CMA: Chromosomal microarray analysis
NGS: Next-generation sequencing
MMS: Microdeletion and microduplication syndromes
PWS: Prader–Willi/Angelman syndrome
