# Supplementary figures and images for: Trends in the prevalence, prenatal diagnosis, and outcomes of births with chromosomal abnormalities: a hospital-based study in Zhejiang Province, China during 2014–2020
Source: Orphanet J Rare Dis. 2022 Dec 22;17:446. doi: 10.1186/s13023-022-02594-1 (PMC9783762; doi:10.1186/s13023-022-02594-1)

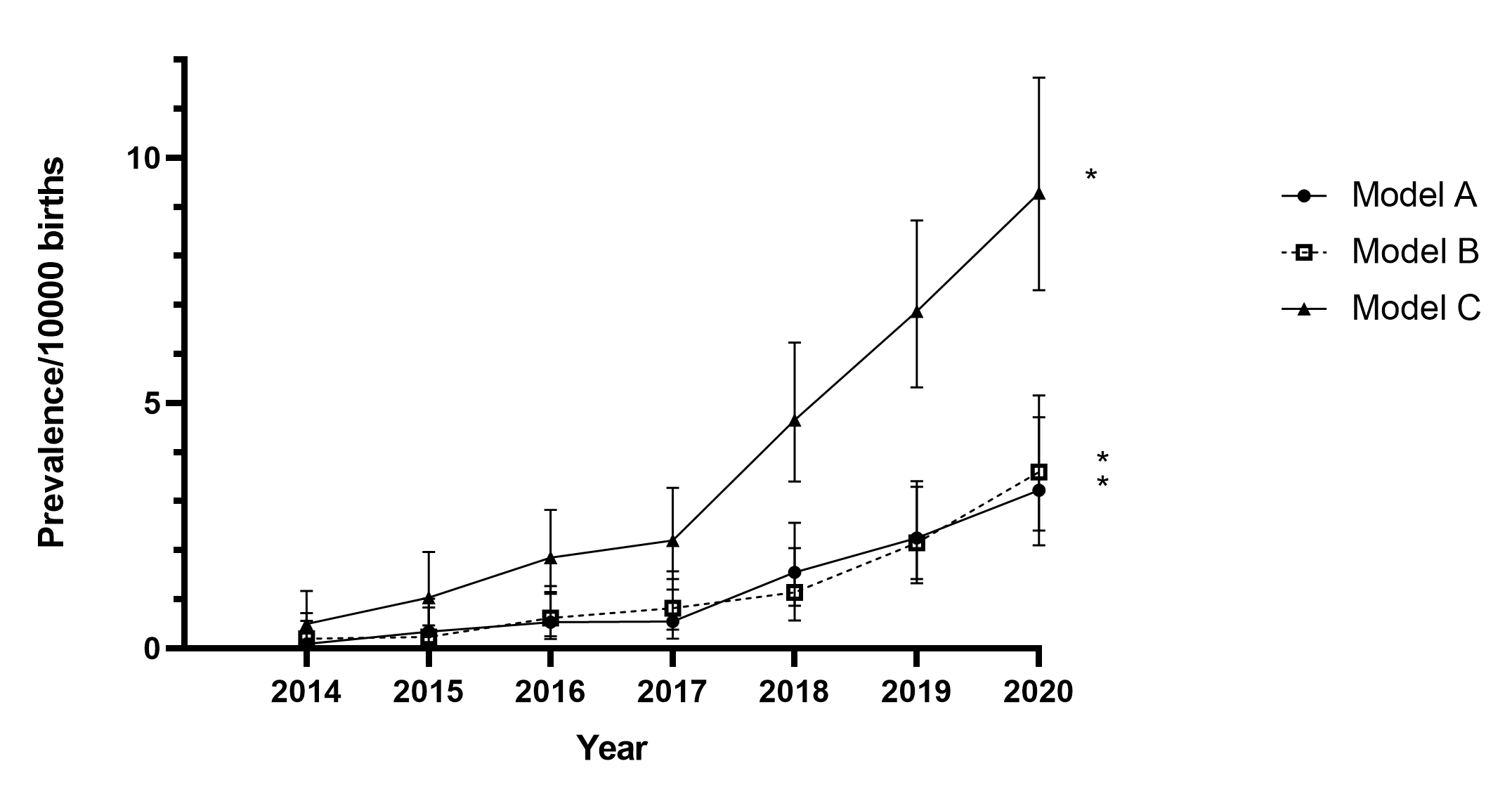

Supplement: Supplementary file 1 — Additional file 1: Fig. S1. Sensitivity analysis for prevalence of microdeletion and microduplication. Sensitivity analysis was carried out for microdeletion and microduplication, since the missing data of CNV size accounted for over 20%. $ Model 1: only cases with CNV < 5 Mb were considered in the analysis. §Model 2: only cases with CNV of 5–10 Mb were in the analysis. ‖Model 3: all cases with CNV <10 Mb as well as cases missing data of CNV size were consideredin the analysis. Significant differences were observed in the Cochran-Armitage Trend Test over the years (Ptrend < 0.001 for all three models of microdeletion and microduplication). [file 13023_2022_2594_MOESM1_ESM.tif]

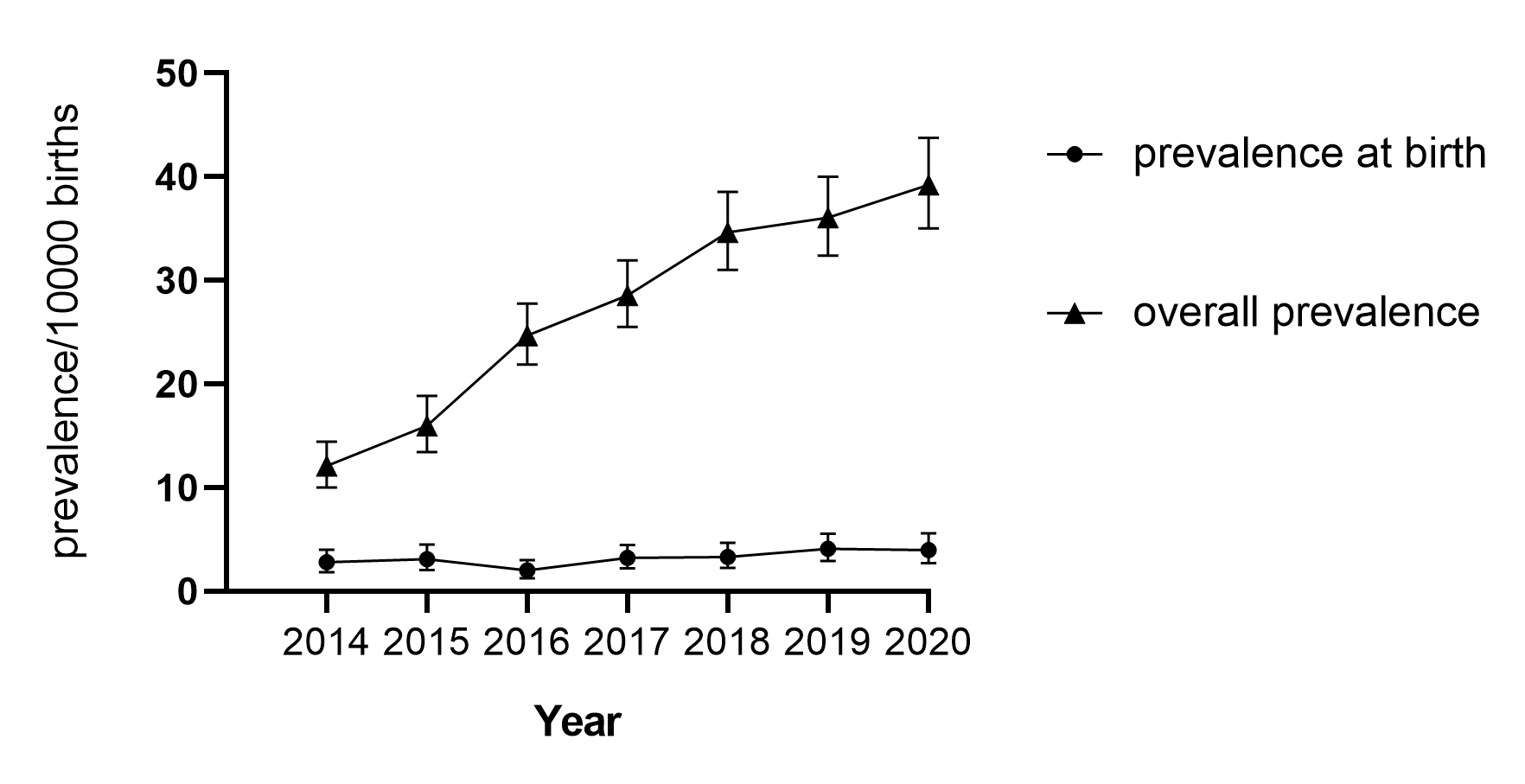

Supplement: Supplementary file 2 — Additional file 2: Fig. S2. Livebirth prevalence of chromosomal abnormalities per 10,000 births in each calendar year, 2014–2020 Figure Legend: Live birth prevalence per 10,000 births was calculated as live births with CA/ total births. Abbreviations: CA, chromosome abnormality. [file 13023_2022_2594_MOESM2_ESM.tif]

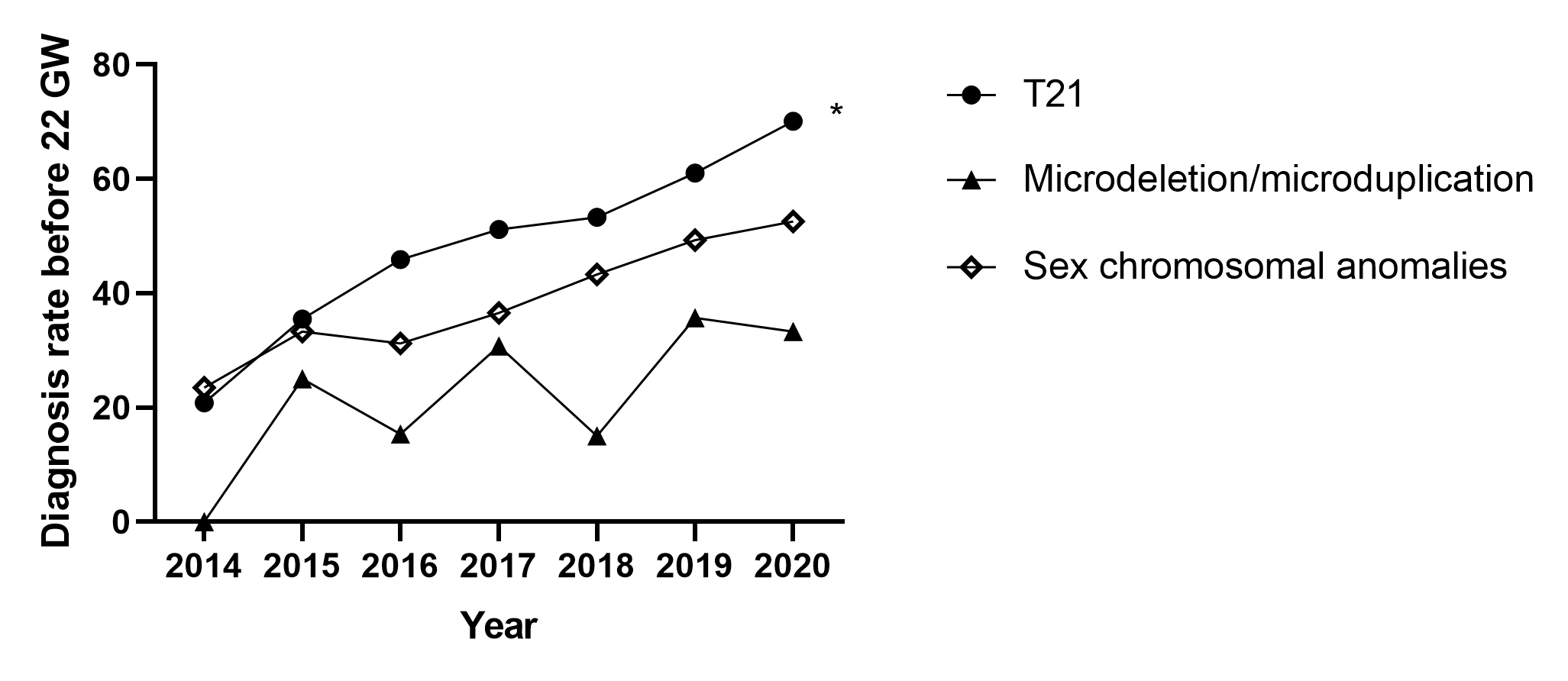

Supplement: Supplementary file 4 — Additional file 4: Fig. S3. Prenatal diagnosis rate before 22 gestational weeks in each calendar year: 2014–2020 *Significant differences were observed by Cochran-Armitage Trend Test over years (Ptrend = 0.003 for trisomy 21, Ptrend = 0.09 for sex chromosome abnormalities) Abbreviations: T21, trisomy 21. [file 13023_2022_2594_MOESM4_ESM.tif]
