# Supplementary material for: Trends in the prevalence, prenatal diagnosis, and outcomes of births with chromosomal abnormalities: a hospital-based study in Zhejiang Province, China during 2014–2020
Source: Orphanet J Rare Dis. 2022 Dec 22;17:446. doi: 10.1186/s13023-022-02594-1 (PMC9783762; doi:10.1186/s13023-022-02594-1)
Supplement: Supplementary file 3 — Additional file 3: Table S1. Prevalence and prenatal diagnosis proportion of chromosomal anomalies by different reporting centres according to surveillance system. Table S2. Baseline characteristics of births with chromosomal abnormalities in Zhejiang Province, 2014–2020. Table S3. Ultrasonographic malformations associated with chromosome abnormalities. [file 13023_2022_2594_MOESM3_ESM.docx]

Supplementary Table 1: Prevalence and prenatal diagnosis proportion of chromosomal anomalies by different reporting centres according to surveillance system

|  | Tertiary hospitals | | | District hospitals | | |
| --- | --- | --- | --- | --- | --- | --- |
|  | No. of births | Prevalence of chromosome abnormalities (per 10,000) | Prenatal diagnosis proportion (%) | No. of births | Prevalence of chromosome abnormalities (per 10,000) | Prenatal diagnosis proportion (%) |
| 2014 | 73040 | 14.38 | 80.00 | 27073 | 5.91 | 81.25 |
| 2015 | 63649 | 19.01 | 89.26 | 23355 | 7.71 | 83.33 |
| 2016 | 89081 | 28.40 | 96.44 | 24721 | 11.33 | 92.86 |
| 2017 | 86094 | 32.41 | 94.27 | 23072 | 14.30 | 96.97 |
| 2018 | 78477 | 37.85 | 97.98 | 18204 | 20.87 | 94.74 |
| 2019 | 80497 | 39.01 | 98.41 | 17101 | 22.22 | 100.00 |
| 2020 | 66220 | 44.10 | 98.63 | 14606 | 17.12 | 96.00 |

Supplementary Table 2：Baseline characteristics of births with chromosomal abnormalities in Zhejiang Province, 2014-2020.

|  | 2014 | 2015 | 2016 | 2017 | 2018 | 2019 | 2020 |
| --- | --- | --- | --- | --- | --- | --- | --- |
| Total cases | 121 | 139 | 281 | 312 | 335 | 352 | 317 |
| Reporting center (1 missing) |  |  |  |  |  |  |  |
| District hospitals | 16 (13.2%) | 18 (12.9%) | 28 (10.0%) | 33 (10.6%) | 38 (11.3%) | 38 (10.8%) | 25 (7.9%) |
| Tertiary hospitals | 105 (86.8%) | 121 (87.1%) | 253 (90.0%) | 279 (89.4%) | 297 (88.7%) | 314 (89.2%) | 292 (92.1%) |
| Maternal Age |  |  |  |  |  |  |  |
| <20 years old | 1(0.8%) | 1 (0.7%) | 0 | 2 (0.6%) | 3 (0.9%) | 0 | 0 |
| 20-24 years old | 11 (9.1%) | 14 (10.1%) | 20 (7.1%) | 30 (9.6%) | 20 (6.0%) | 27 (7.7%) | 21 (6.6%) |
| 25-29 years old | 36 (29.8%) | 49 (35.3%) | 81 (28.8%) | 79 (25.3%) | 102 (30.4%) | 92 (26.1%) | 103 (32.5%) |
| 30-34 years old | 40 (33.1%) | 27 (19.4%) | 61 (21.7%) | 69 (22.1%) | 91 (27.2%) | 119 (33.8%) | 102 (32.2%) |
| ≥35 years old | 33 (27.3%) | 48 (34.5%) | 119 (42.3%) | 132 (42.3%) | 119 (35.5%) | 114 (32.4%) | 91 (28.7%) |
| Maternal Education (1 missing) | | | | | | | |
| illiterate or primary school | 3 (2.5%) | 4 (2.9%) | 8 (2.8%) | 10 (3.2%) | 13 (3.9%) | 11 (3.2%) | 10 (3.1%) |
| junior high school | 19 (15.7%) | 31 (22.3%) | 35 (12.5%) | 60 (19.2%) | 45 (13.4%) | 46 (13.1%) | 40 (12.6%) |
| senior high school | 20 (16.5%) | 21 (15.1%) | 31 (11.0%) | 46 (14.7%) | 45 (13.4%) | 43 (12.2%) | 35 (11.0%) |
| undergraduate or higher | 79 (65.3%) | 83 (59.7%) | 206 (73.3%) | 196 (62.8%) | 232 (69.3%) | 252 (71.6%) | 232 (73.2%) |
| Maternal Parity |  |  |  |  |  |  |  |
| primiparas | 95 (78.5%) | 105 (75.5%) | 216 (76.9%) | 240 (76.9%) | 260 (77.6%) | 273 (77.6%) | 246 (77.6%) |
| multiparas | 26 (21.5%) | 34 (24.5%) | 65 (23.1%) | 72 (23.1%) | 75 (22.4%) | 69 (22.4%) | 71 (22.4%) |
| Past history of delivering babies with birth defects | 5 (4.1%) | 3 (2.2%) | 2 (0.7%) | 7 (2.2%) | 5 (1.5%) | 9 (2.6%) | 18 (5.7%) |
| Delivery time |  |  |  |  |  |  |  |
| <22 gestational weeks | 1 (0.8%) | 7 (5.0%) | 24 (8.5%) | 21 (6.7%) | 29 (8.7%) | 37 (10.5%) | 42 (13.2%) |
| ≥22 gestational weeks | 120 (99.2%) | 132 (95.0%) | 257 (91.5%) | 291 (93.3%) | 306 (91.3%) | 315 (89.5%) | 275 (86.8%) |
| Prenatal outcomes (1 missing) |  |  |  |  |  |  |  |
| Survived births | 26 (21.5%) | 22 (15.8%) | 21 (7.5%) | 31 (9.9%) | 29 (8.7%） | 37 (10.5%) | 32 (10.1%) |
| TOPFA | 93 (76.9%) | 112 (80.6%) | 258 (91.8%) | 277 (88.8%) | 303 (90.4%) | 312 (88.6%) | 285 (89.9%) |
| Fetal loss or stillbirths (except TOPFA) | 0 | 3 (2.2%) | 0 | 3 (1.0%) | 3 (0.9%) | 2 (0.6%) | 0 |
| Newborn deaths | 2 (1.7%) | 2 (1.4%) | 2 (0.7%) | 1 (0.3%) | 0 | 1 (0.3%) | 0 |

Abbreviations: TOPFA, termination of pregnancy because of fetal anomalies.

Supplementary Table 3: Ultrasonographic malformations associated with chromosome abnormalities

| Types of associated structural anomalies | Total cases | N in microdeletion/microduplication | N in SCA | N in aneuploidies |
| --- | --- | --- | --- | --- |
| cardiac anomalies | 112 | 19 | 8 | 81 |
| cleft lip/cleft palate | 23 | 5 | 1 | 17 |
| malformation of kidney | 22 | 13 | 4 | 3 |
| absence of a nasal bone | 18 | 4 | 0 | 14 |
| nuchal cystic hygroma | 16 | 3 | 8 | 3 |
| neuroanatomic anomalies ^a^ | 16 | 1 | 1 | 14 |
| atresia of digestive tract | 13 | 1 | 0 | 11 |
| clubfoot | 12 | 3 | 4 | 4 |
| polydactyly/syndactyly | 11 | 2 | 2 | 7 |
| congenital hydrocephalus | 6 | 1 | 1 | 4 |
| omphalocele and gastroschisis | 6 | 2 | 0 | 4 |

1. Except hydrocephalus
